# Oxidative Metabolites of Diisononyl Phthalate as Biomarkers for Human Exposure Assessment

**DOI:** 10.1289/ehp.8865

**Published:** 2006-03-27

**Authors:** Manori J. Silva, John A. Reidy, James L. Preau, Larry L. Needham, Antonia M. Calafat

**Affiliations:** Division of Laboratory Sciences, National Center for Environmental Health, Centers for Disease Control and Prevention, Atlanta, Georgia, USA

**Keywords:** biomarkers, diisononyl phthalate, DINP, MINP, monoisononyl phthalate, oxidative metabolites

## Abstract

Diisononyl phthalate (DINP) is a complex mixture of predominantly nine-carbon branched-chain dialkyl phthalate isomers. Similar to di(2-ethylhexyl) phthalate, a widely used phthalate, DINP causes antiandrogenic effects on developing rodent male fetuses. Traditionally, assessment of human exposure to DINP has been done using monoisononyl phthalate (MINP), the hydrolytic metabolite of DINP, as a biomarker. However, MINP is only a minor urinary metabolite of DINP. Oxidative metabolites, including mono(carboxyisooctyl) phthalate (MCIOP), mono(oxoisononyl) phthalate (MOINP), and mono(hydroxyisononyl) phthalate (MHINP) are the major urinary metabolites in DINP-dosed rats. The urinary concentrations of MINP, MCIOP, MOINP, and MHINP were measured in 129 adult anonymous human volunteers with no known exposure to DINP. Although MINP was not present at detectable levels in any of the samples analyzed, MCIOP, MHINP, and MOINP were detected in 97, 100, and 87% of the urine samples at geometric mean levels equal to 8.6, 11.4, and 1.2 ng/mL, respectively. The concentrations of all three oxidative metabolites were highly correlated with each other (*p* < 0.0001), which confirms a common precursor. MCIOP was excreted predominantly as a free species, whereas MOINP was excreted mostly in its glucuronidated form. The percentage of MHINP excreted either glucuronidated or in its free form was similar. The significantly higher frequency of detection and urinary concentrations of oxidative metabolites than of MINP suggest that these oxidative metabolites are better biomarkers of exposure assessment of DINP than is MINP. Therefore, we concluded that the prevalence of human exposure to DINP is underestimated by using MINP as the sole DINP urinary biomarker.

Diisononyl phthalate (DINP) is a complex mixture of branched-chain dialkyl phthalate isomers, predominantly containing nine carbons in the alkyl chain. DINP is used primarily as a plasticizer in polyvinyl chloride plastics (Abe et al. 2003) and is widely used in automotives, building materials, consumer products, and toys [Center for the Evaluation of Risks to Human Reproduction (CERHR) 2000; Kavlock et al. 2002].

In rats, DINP shows antiandrogenic activity (Gray et al. 2000). Specifically, nipple retention and testis atrophy after perinatal exposure to 750 mg/kg DINP have been observed in male rats (Gray et al. 2000). It appears that DINP, like di(2-ethylhexyl) phthalate (DEHP), a widely used phthalate, alters sexual differentiation of the male rat by inhibiting testicular testosterone synthesis (Gray et al. 2000). Furthermore, evidence has shown that oral exposure to DINP causes liver and kidney toxicity in adult rats and mice (Kaufmann et al. 2002). The liver effects are generally consistent with those associated with peroxisome proliferation (Kaufmann et al. 2002).

DINP biomonitoring to measure exposure in humans is of interest because of the potential adverse health effects of DINP. More important, children may be exposed to higher levels of DINP than adults because infants and small children mouth toys and other articles that can contain DINP (Kavlock et al. 2002). Because DINP is not covalently bound to the plastics, it can migrate into saliva and be swallowed (Kavlock et al. 2002). In previous studies the hydrolytic monoester of DINP, monoisononyl phthalate (MINP), has been used for human exposure assessment of DINP [Centers for Disease Control and Prevention (CDC) 2005; Silva et al. 2004a]. However, the frequency of detection of MINP was very low compared with other phthalate metabolites. The low frequency of detection of MINP in human populations may be attributable, at least in part, to the fact that MINP further metabolizes to form oxidative metabolites before being excreted in urine. Although the metabolism of DEHP (Albro 1986; Koch et al. 2004, 2005a; Silva et al. 2006b, 2006c) and di-*n*-octyl phthalate (DnOP) (Albro and Moore 1974; Calafat et al. 2006; Silva et al. 2005) in rodents and humans is relatively well known, the metabolism of DINP has been less studied (McKee et al. 2002).

In rodents, MINP was found to metabolize to unidentified oxidative products (McKee et al. 2002). Recently, several urinary oxidative metabolites of DINP were identified and detected at much higher concentrations than MINP in DINP-dosed rats (Silva et al. 2006a). It was postulated that these metabolites could be used as biomarkers of exposure to DINP in humans (Silva et al. 2006a). In this study, MINP and three of these oxidative metabolites, mono(carboxyisooctyl) phthalate (MCIOP), mono(hydroxyisononyl) phthalate (MHINP), and mono(oxoisononyl) phthalate (MOINP) (Figure 1), were measured in 129 human urine samples from adults with no known exposure to DINP. As in rodents, in this group of adults the frequency and the magnitude of detection were significantly higher for the oxidative metabolites than for MINP.

## Materials and Methods

We purchased mono(2-ethyl-5-hydroxyhexyl) phthalate (MEHHP), mono(2-ethyl-5-oxohexyl) phthalate (MEOHP), mono(2-ethylhexyl) phthalate (MEHP), mono-3-methyl-5-dimethylhexyl phthalate (MINP), ^13^C_4_-MEHP, ^13^C_4_-MEHHP, ^13^C_4_-MEOHP, ^13^C_4_-MINP, and ^13^C_4_-4-methylumbel-liferone (^13^C_4_-MeUmb) from Cambridge Isotopes Laboratories Inc. (Andover, MA). Mono(2-ethyl-5-carboxypentyl) phthalate (MECPP) and D_4_ -MECPP were gifts from J. Angerer (University of Erlangen, Nuremberg, Germany). HPLC-grade acetonitrile and water were purchased from Tedia (Fairfield, OH), and MeUmb and its glucuronide (MeUmb-glu) were purchased from Sigma Chemical Co. (St. Louis, MO). β-Glucuronidase (*Escherichia coli*-K12) was purchased from Roche Biomedical (Mannheim, Germany). Stock solutions of standards (MEHP, MEOHP, MEHHP, and MeUmb) and internal standards (^13^C_4_-MEHP, ^13^C_4_-MEHHP, ^13^C_4_-MEOHP, and ^13^C_4_-MeUmb) were prepared in acetonitrile. ^13^C_4_-MEOHP was used as the internal standard for MOINP, D_4_-MECPP was used as the internal standard for MCIOP, and ^13^C_4_-MEHHP was used as the internal standard for MHINP.

Urine samples were collected by each study participant directly into a phthalate-free prescreened urine cup. The analytical method for measuring DINP oxidative metabolites in urine was adapted from previously published methods (Blount et al. 2000; Silva et al. 2003, 2004b). Briefly, the urine samples (1 mL) were spiked with an internal standard solution containing ^13^C_4_-MEHP, ^13^C_4_-MEOHP, ^13^C_4_-MEHHP, D_4_-MECPP, ^13^C_4_-MINP, and 4-MeUmb. MeUmb-glu was added to evaluate the completion of the deglucuronidation reaction with β-glucuronidase. Phthalate monoester metabolites were extracted by automated solid-phase extraction (SPE) using a commercial SPE system (Zymark Corp., Hopkinton, MA) after enzymatic hydrolysis. The metabolites in the urine extract were chromatographically resolved by high-performance liquid chromatography (HPLC) using a Surveyor HPLC system (ThermoFinnigan, San Jose, CA) equipped with a Betasil phenyl HPLC column (3 μm, 100 mm × 2.1 mm; ThermoHypersil-Keystone, Bellefonte, PA) using a nonlinear water:acetonitrile solvent gradient. The metabolites were detected by negative ion electrospray ionization tandem mass spectrometry using a ThermoFinnigan TSQ Quantum triple quadrupole mass spectrometer (ThermoFinnigan). For the measurement of the unconjugated metabolites, we eliminated treatment with β-glucuronidase. Under our experimental conditions, the isomeric metabolites of DINP were not chromatographically resolved and eluted as broad peaks. The entire area under the peak encompassing all isomers was integrated for quantification. The limits of detection (LODs) were 0.25 ng/mL for MOINP, MHINP, and MCIOP. The LOD for MINP was 0.36 ng/mL. Oxidative metabolism is an enzymatically mediated reaction. Therefore, oxidative metabolites cannot result from potential contamination with DINP during sampling, storage, or analysis.

Statistical analysis of the data was performed using the Statistical Analysis System (SAS) software (SAS Institute Inc., Cary, NC). Samples with values below the LOD were assigned a concentration equal to the LOD divided by the square root of 2 for the statistical analyses (Hornung and Reed 1990). Statistical significance was set at *p* < 0.05.

### Subjects

The urine samples analyzed for this study were collected specifically for analysis of phthalate metabolites in 2005 from a demographically diverse group of 129 U.S. adults of both sexes with no documented exposure to DINP. No personal information from the subjects was available. Samples were collected between 0800 hr and 1700 hr and were not necessarily first morning voids. The study protocol was reviewed and approved by the CDC Human Subjects Institutional Review Board. A waver for informed consent for this project was requested under 45 CFR 46.116(*d*) (Code of Federal Regulations 2005).

## Results and Discussion

Although DINP is a less potent inducer of peroxisomal proliferation than DEHP (McKee et al. 2000), DINP exerts antiandrogenic effects similar to that of DEHP in DINP-dosed rats (Gray et al. 2000). The effects of DINP exposure in humans are not currently known.

Phthalates with long alkyl side chains, such as DEHP and DnOP, metabolize extensively before being excreted in urine both in rodents and humans (Albro 1986; Albro and Moore 1974; Koch et al. 2004, 2005b; Silva et al. 2005). Similarly, in rats administered DINP, some MINP was excreted in urine, but oxidative metabolites of MINP, MHINP, MCIOP, and MOINP were excreted as the major urinary metabolites (Silva et al. 2006a).

We measured the urinary concentrations of MINP, MHINP, MCIOP, and MOINP in 129 human adults. We observed a wide range of exposures to DINP (Table 1). MHINP was present in all samples tested at concentrations ranging from 1.4 to 202.7 ng/mL, with 5% of the samples having > 43.7 ng/mL (Table 1). Similarly, MCIOP was detected in 97% of the samples tested at levels ranging from < LOD to 310.8 ng/mL. MOINP was detected in 87% of the samples at levels ranging from < LOD to 201.7 ng/mL (Table 1). The geometric mean concentrations of MHINP, MCIOP, and MOINP were 11.4, 8.6, and 1.2 ng/mL, respectively. Interestingly, we did not detect MINP in any of the samples analyzed.

**Figure 2:**
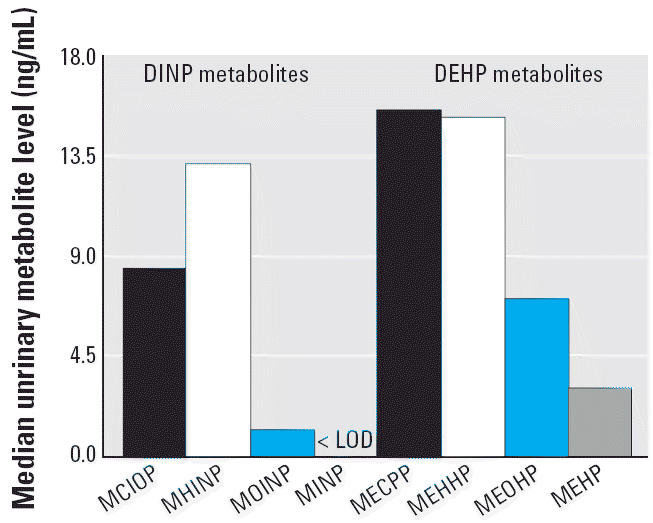
Median levels of DINP and DEHP metabolites in a group of 129 U.S. adults. For concentrations < LOD, a value of LOD/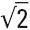 was used for the statistical computations.

In rats dosed with DINP, the major metabolite excreted in urine was MCIOP (Silva et al. 2006a). By contrast, in this study population, MHINP was excreted as the major urinary metabolite (Table 1). Although three oxidative metabolites of DINP were present in all samples analyzed, the urinary concentrations of these metabolites were lower than the structurally related DEHP metabolites MEHHP, MEOHP, and MECPP, also measured in this population (Silva et al. 2006b) (Figure 2). In humans, the urinary concentrations of MEHHP, MEOHP, MECPP, and MEHP represent about 75% of the DEHP dose (Koch et al. 2004, 2005b). The fraction of DINP excreted in urine as MHINP, MOINP, MCIOP, and MINP is not presently known. Based on similar physicochemical properties and metabolism between DINP and DEHP (Koch et al. 2004, 2005b; Silva et al. 2006b, 2006c), the lower concentrations of DINP oxidative metabolites than of DEHP oxidative metabolites in this group of adults suggest that environmental exposures to DINP may be lower than the exposure to DEHP. However, because DINP is a mixture of isomers, it is also possible that the prevalence of exposure to DINP is underestimated by measuring only these three oxidative metabolites. Furthermore, the elimination half-life of the oxidative metabolites of DINP is presently unknown. Therefore, the differences in urinary concentrations observed among DINP oxidative metabolites and their DEHP counterparts may also reflect differences in toxicokinetic parameters.

**Figure 3:**
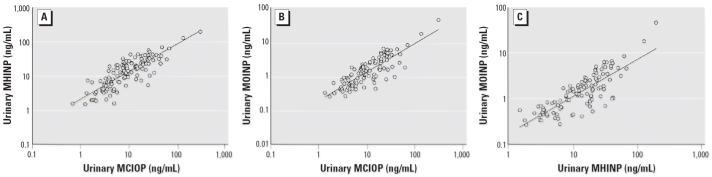
Correlation analyses of urinary MCIOP, MHINP, and MOINP. *R* represents Pearson correlation coefficient. Levels below the LOD were excluded in the analysis: (*A*) *R* = 0.83, *p* < 0.0001; (*B*) *R* = 0.76, *p* < 0.0001; (*C*) *R* = 0.73, *p* < 0.0001.

Because all three DINP metabolites result from the same parent compound, their urinary concentrations were highly correlated with each other, with correlation coefficients varying from 0.73 to 0.83 (*p* < 0.001; Figure 3), similar to previous findings regarding DEHP metabolites (Barr et al. 2003; Kato et al. 2004; Koch et al. 2004, 2005b).

**Figure 4:**
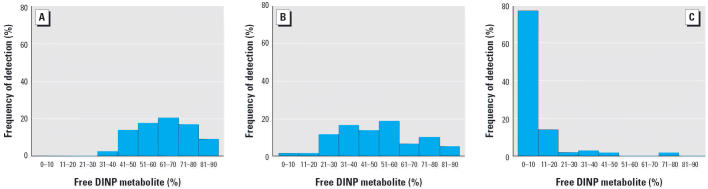
Frequency of detection of free urinary DINP oxidative metabolites: (*A*) MCIOP, (*B*) MHINP, and (*C*) MOINP.

**Figure 5:**
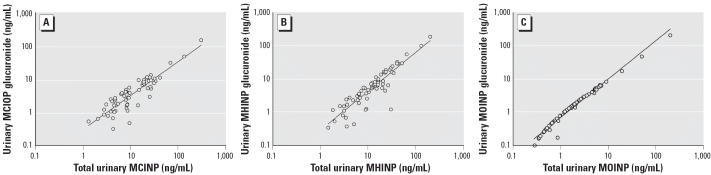
Correlation analyses of the glucuronide-conjugated DINP metabolites and the total (free and glucuronidated). Levels < LOD were eliminated in the graphical representations. (*A*) *R* = 0.90, *p* < 0.0001; (*B*) *R* = 0.90, *p* < 0.0001; (*C*) *R* = 0.98, *p* < 0.0001.

Glucuronidation not only facilitates urinary excretion of phthalate metabolites but may also reduce their potential biological activity if the putative biologically active species is the free metabolite. We measured both total and free urinary concentrations of MHINP, MOINP, and MCIOP and found that although MCIOP mostly excreted in its free form, MOINP excreted mostly glucuronidated. The percentage of MHINP excreted either as a conjugate or free form was similar (Figure 4). Furthermore, the concentration of the glucuronidated form of the metabolites increased with increasing levels of the total metabolite concentrations, indicating the absence of enzyme saturation at environmental exposure levels (Figure 5).

In summary, we measured the urinary concentrations of three oxidative metabolites of DINP (MCIOP, MHINP, and MOINP) and the hydrolytic metabolite MINP in 129 anonymous adults. The oxidative metabolites were present in all samples tested, and their urinary concentrations were highly correlated with each other. By contrast, the hydrolytic monoester MINP was not detected in any of the samples. The most abundant DINP urinary metabolites were the ω and ω^−1^ oxidative metabolites, MCIOP and MHINP, respectively. MCIOP was excreted in urine predominantly in its free form, whereas MOINP was excreted glucuronidated. The significantly higher frequency of detection and urinary levels of oxidative metabolites than of MINP confirm the validity of these oxidative metabolites as biomarkers for DINP exposure assessment. More important, these data suggest that exposure to DINP is widespread and that it has been underestimated by using MINP as the sole DINP urinary biomarker.

## Correction

In Figure 5, values for urinary MOINP glucuronide on the *y*-axis have been modified from the original manuscript published online. The corrected values are 0.1, 1, 10, 100, and 1,000. The original values were 0.1, 1, 10, and 100.

## Figures and Tables

**Figure 1:**
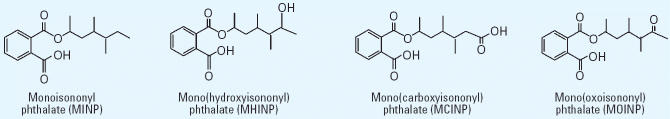
DINP metabolites proposed as biomarkers for exposure assessment to DINP in humans. Structures shown are for only one of the potential isomers.

**Table 1:** Urinary levels (ng/mL) of DINP metabolites in a group of 129 U.S. adults.

		Selected percentiles							
Urinary DINP metabolite^a^	*n*	10th	25th	50th	75th	90th	95th	Geometric mean^b^	Frequency of detection (%)
MCIOP									
Total	129	2.0	3.9	8.4	18.3	27.3	46.2	7.8	97
Free^c^	82	2.0	2.9	5.1	11.6	22.8	1.5	6.1	98
MHINP									
Total	129	2.6	5.4	13.2	23.2	40.2	43.7	11.4	100
Free^c^	82	1.8	2.9	5.8	9.1	15.5	20.1	5.4	100
MOINP									
Total	129	< LOD	0.5	1.2	2.4	5.0	6.6	1.2	87
Free^c^	82	< LOD	< LOD	< LOD	0.3	0.7	1.3	NA	30
MINP									
Free^c^	129	< LOD	< LOD	< LOD	< LOD	< LOD	< LOD	< LOD	0
Total	82	< LOD	< LOD	< LOD	< LOD	< LOD	< LOD	< LOD	0

NA, applicable: the geometric mean was calculated only if the frequency of detection was ≥ 60%.

a D_4_-MECPP was used as the internal standard for MCIOP. ^13^C_4_-MEOHP was used as the internal standard for MOINP. ^13^C_4_-MEHHP and ^13^C_4_-MINP were used as the internal standards for MHINP and MINP, respectively.

b LOD/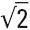 was used for the statistical computations if the concentration was below the LOD. LODs were 0.36 ng/mL (MINP) and 0.25 ng/mL (MCIOP, MHINP, and MOINP).

c Only 82 samples were available in sufficient quantities to determine the concentrations of free metabolites.

## References

[b1-ehp0114-001158] Abe Y, Sugita T, Wakui C, Niino T, Yomota C, Ishiwata H (2003). Material labeling of soft plastic toys and plasticizers in polyvinyl chloride
products. J Food Hygienic Soc Jpn.

[b2-ehp0114-001158] Albro PW (1986). Absorption, metabolism, and excretion of di(2-ethylhexyl) phthalate by
rats and mice. Environ Health Perspect.

[b3-ehp0114-001158] Albro PW, Moore B (1974). Identification of the metabolites of simple phthalate diesters in rat urine. J Chromatogr.

[b4-ehp0114-001158] Barr DB, Silva MJ, Kato K, Reidy JA, Malek NA, Hurtz D (2003). Assessing human exposure to phthalates using monoesters and their oxidized
metabolites as biomarkers. Environ Health Perspect.

[b5-ehp0114-001158] Blount BC, Milgram KE, Silva MJ, Malek NA, Reidy JA, Needham LL (2000). Quantitative detection of eight phthalate metabolites in human urine using
HPLC-APCI-MS/MS. Anal Chem.

[b6-ehp0114-001158] Calafat AM, Silva MJ, Reidy JA, Gray LE, Samandar E, Preau JLJ (2006). Mono-3-carboxypropyl phthalate, a metabolite of di-*n*-octyl phthalate. J Toxicol Environ Health A.

[b7-ehp0114-001158] CDC 2005. Third National Report on Human Exposure to Environmental Chemicals. Atlanta, GA:Centers for Disease Control and Prevention, National Center for Environmental Health, Division of Laboratory Sciences. Available: http://www.cdc.gov/exposurereport/3rd/pdf/thirdreport.pdf [accessed 11 August 2005].

[b8-ehp0114-001158] CERHR (Center for the Evaluation of Risks to Human Reproduction) 2000. NTP-CERHR Expert Panel Report on Di-isononyl Phthalate. Research Triangle Park, NC:National Toxicology Program, U.S. Department of Health and Human Services. Available: http://cerhr.niehs.nih.gov/news/index.html [accessed 26 July 2004].

[b9-ehp0114-001158] Code of Federal Regulations 2005. General Requirements for Informed Consent. 45 CFR 46.116(*d*).

[b10-ehp0114-001158] Gray LE, Ostby J, Furr J, Price M, Veeramachaneni DNR, Parks L (2000). Perinatal exposure to the phthalates DEHP, BBP, and DINP, but not DEP, DMP, or
DOTP, alters sexual differentiation of the male rat. Toxicol Sci.

[b11-ehp0114-001158] Hornung RW, Reed LD (1990). Estimation of average concentration in the presence of nondetectable values. Appl Occup Environ Hyg.

[b12-ehp0114-001158] Kato K, Silva MJ, Reidy JA, Hurtz D, Malek NA, Needham LL (2004). Mono(2-ethyl-5-hydroxyhexyl) phthalate and mono-(2-ethyl-5-oxohexyl) phthalate
as biomarkers for human exposure assessment to di-(2-ethylhexyl) phthalate. Environ Health Perspect.

[b13-ehp0114-001158] Kaufmann W, Deckardt K, McKee RH, Butala JH, Bahnemann R (2002). Tumor induction in mouse liver: di-isononyl phthalate acts via peroxisome
proliferation. Regul Toxicol Pharmacol.

[b14-ehp0114-001158] Kavlock R, Boekelheide K, Chapin R, Cunningham M, Faustman E, Foster P (2002). NTP Center for the Evaluation of Risks to Human Reproduction: phthalates
expert panel report on the reproductive and developmental toxicity of
di-isononyl phthalate. Reprod Toxicol.

[b15-ehp0114-001158] Koch HM, Bolt HM, Angerer J (2004). Di(2-ethylhexyl)phthalate (DEHP) metabolites in human urine and serum after
a single oral dose of deuterium-labelled DEHP. Arch Toxicol.

[b16-ehp0114-001158] Koch HM, Bolt HM, Preuss R, Angerer J (2005a). New metabolites of di(2-ethylhexyl)phthalate (DEHP) in human urine and
serum after single oral doses of deuterium-labelled DEHP. Arch Toxicol.

[b17-ehp0114-001158] Koch HM, Bolt HM, Preuss R, Eckstein R, Weisbach V, Angerer J (2005b). Intravenous exposure to di(2-ethylhexyl)phthalate (DEHP): metabolites of
DEHP in urine after a voluntary platelet donation. Arch Toxicol.

[b18-ehp0114-001158] McKee RH, El Hawari M, Stoltz M, Pallas F, Lington AW (2002). Absorption, disposition and metabolism of di-isononyl phthalate (DINP) in
F-344 rats. J Appl Toxicol.

[b19-ehp0114-001158] McKee RH, Przygoda RT, Chirdon MA, Engelhardt G, Stanley M (2000). Di(isononyl) phthalate (DINP) and di(isodecyl) phthalate (DIDP) are not
mutagenic. J Appl Toxicol.

[b20-ehp0114-001158] Silva MJ, Barr DB, Reidy JA, Malek NA, Hodge CC, Caudill SP (2004a). Urinary levels of seven phthalate metabolites in the US population from
the National Health and Nutrition Examination Survey (NHANES) 1999–2000. Environ Health Perspect.

[b21-ehp0114-001158] Silva MJ, Kato K, Gray EL, Wolf C, Needham LL, Calafat AM (2005). Urinary metabolites of di-*n*-octyl phthalate in rats. Toxicology.

[b22-ehp0114-001158] Silva MJ, Kato K, Wolf C, Samandar E, Silva SS, Gray LE (2006a). Urinary biomarkers of di-isononyl phthalate in rats. Toxicology.

[b23-ehp0114-001158] Silva MJ, Malek NA, Hodge CC, Reidy JA, Kato K, Barr DB (2003). Improved quantitative detection of 11 urinary phthalate metabolites in
humans using liquid chromatography-atmospheric pressure chemical ionization
tandem mass spectrometry. J Chromatogr B.

[b24-ehp0114-001158] Silva MJ, Reidy JA, Samandar E, Preau JLJ, Needham LL, Calafat AM (2006b). Measurement of eight urinary metabolites of di(2-ethylhexyl) phthalate
as biomarkers for human exposure assessment. Biomarkers.

[b25-ehp0114-001158] Silva MJ, Samandar E, Preau JLJ, Needham LL, Calafat AM (2006c). Urinary oxidative metabolites of di(2-ethylhexyl) phthalate in humans. Toxicology.

[b26-ehp0114-001158] Silva MJ, Slakman AR, Reidy JA, Preau JL, Herbert AR, Samandar E (2004b). Analysis of human urine for fifteen phthalate metabolites using automated
solid-phase extraction. J Chromatogr B.

